# *Leishmania* spp. Proteome Data Sets: A Comprehensive Resource for Vaccine Development to Target Visceral Leishmaniasis

**DOI:** 10.3389/fimmu.2014.00260

**Published:** 2014-06-10

**Authors:** Toni Aebischer

**Affiliations:** ^1^Agents of Mycoses, Parasitoses and Mycobacterioses, Robert Koch-Institut, Berlin, Germany

**Keywords:** proteome, T cell antigen receptor, vaccine, kinetoplastida, major histocompatibility complex antigens

## Abstract

Visceral leishmaniasis is a neglected infectious disease caused primarily by *Leishmania donovani* and *Leishmania infantum* protozoan parasites. A significant number of infections take a fatal course. Drug therapy is available but still costly and parasites resistant to first line drugs are observed. Despite many years of trial no commercial vaccine is available to date. However, development of a cost effective, needle-independent vaccine remains a high priority. Reverse vaccinology has attracted much attention since the term has been coined and the approach tested by Rappuoli and colleagues. This *in silico* selection of antigens from genomic and proteomic data sets was also adapted to aim at developing an anti-*Leishmania* vaccine. Here, an analysis of the efforts is attempted and the challenges to be overcome by these endeavors are discussed. Strategies that led to successful identification of antigens will be illustrated. Furthermore, these efforts are viewed in the context of anticipated modes of action of effective anti-*Leishmania* immune responses to highlight possible advantages and shortcomings.

## Introduction

A cure or effective prophylaxis for visceral leishmaniasis (VL) also known as Kala azar is a prioritized objective in global efforts directed toward improving the situation for people at risk of and patients suffering from *Leishmania*-infections (1, 2). The problem of VL is grave as it is thought to be second only to malaria in terms of fatal infections (3). Therapy is one option to help the individual patient but on its own is unlikely to offer a lasting solution to manage the public health problem because of emerging resistance to available drugs (4). Vaccines are therefore considered a desirable, cost effective strategy complement (5).

There is encouraging evidence that vaccination against VL should be possible. Immunity is thought to depend on a protective cellular immune response requiring CD4 as well as CD8 T cells that activate leishmanicidal mechanisms in host phagocytes (6, 7) since their suppression correlates with disease (8, 9). Epidemiological data suggest that the majority of infections are in fact controlled and do not lead to disease. For example, the KalaNet study reported an estimate of only 1 in 10 infections leading to disease in India and Nepal where more than 50% of globally recorded fatal VL cases occur (10, 11). In addition, there is the paradigmatic example of lifelong protection against cutaneous leishmaniasis through the century old practice of Leishmanization. This deliberate infection of a non-immune person with virulent parasites (12) has been implemented in the immunization programs of soldiers of several armies in the Middle East but has been discontinued because of the risk of uncontrolled disease in a fraction of vaccines (13) and problems with vaccine strain stability (14). The protective efficacy against subsequent infection afforded by a healed primary infection due to Leishmanization in the majority of cases fostered the development of attenuated live parasites (15–18) or parasites not pathogenic to humans (19) as vaccines also against VL. This approach works remarkably well in rodent models of disease and may be a very promising approach to control VL where this is fueled by a zoonotic cycle.

For human use, subunit vaccines based on selected parasite antigens, however, would offer a more defined and more stable alternative (20). But, major obstacles to their successful development exist and these are on the one hand the identification of the most effective antigens and on the other hand their formulation. Formulation relates to selecting adjuvants and/or delivery systems such as recombinant viruses (2, 21, 22) or bacteria (23, 24) and exploitation or engineering of immune-modulating agents and properties to induce protective antigen-specific CD4 and CD8 T cells. Although our understanding of what makes a protective response in humans remains sketchy (8, 9, 25), there is no reason to object to the idea that this can be achieved through vaccine formulation if selected *Leishmania*-antigens were fit for purpose.

In the post-genomic era, the approach to antigen selection and vaccine development has been revolutionized. The term reverse vaccinology has been coined by Rappuoli and colleagues (26) at the turn of the millennium to designate the process. The idea is simple and is about exploiting genomic and other -omics data sets to filter out relevant gene products *in silico*. Selection proceeds through an algorithm that is developed “backwards” starting from a known or anticipated mode of action of the vaccine. This has been impressively successful for the development of novel anti-Meningococcal Serotype B vaccines because (a) the mode of action was known and allowed to develop a straight forward *in vitro* screening assay based on lysis-mediating antibodies and (b) this assay was scalable and had high throughput capacity (26, 27). The Reverse Vaccinology approach has also been adapted to identify potential vaccine protein antigens against leishmaniasis and the combined search terms “reverse vaccinology” and “*leishmania*” retrieve five publications from PubMed as of March 10th 2014 (24, 28–32). Reverse Vaccinology when adapted to VL will aim at identifying vaccine antigens that induce protective CD4 and CD8 T cells (24, 28, 30).

In the following, I will try to critically assess the adaptation of the Reverse Vaccinology approach to the development of an anti-VL vaccine. However, before doing so, I will summarize in a bullet point way features of the cell biology of *Leishmania*-infection and of MHC class I and II dependent antigen-presentation in the context of this infection. The aim is to distil scenarios that allow identification of process-relevant steps through which reverse vaccinology may be improved. The reader will quickly note that this comes at a price. This is the deliberate simplification of our understanding of the parasite’s intracellular life style.

## Bullet Point Style Summary of the Cell Biology of *Leishmania* spp. Infections

- Disease-causing *Leishmania* replicate in the form of amastigotes in a membrane-delimited intracellular habitat of host phagocytes (33).
- The habitat has the characteristics of a late endosome/early lysosome, i.e., a relatively low pH with numerous proteases such as cathepsins and other hydrolases present (34, 35).
- The parasites’ habitat is in communication with the host cell’s endocytic compartments via fusion and fission of vesicles (36, 37).
- Parasite protein secretion can occur via the classical, signal peptide-dependent pathways or, as recently favored, via the release of exosomes (38, 39).

## Bullet Point Style Summary of Antigen-Presentation by *Leishmania*-Infected Host Cells

- Parasite proteins are processed for presentation by proteolysis inside vesicles and it is within a vesicular compartment that peptides form complexes with MHC class I and II histocompatibility antigens (37, 40).
- The so-called cross-presentation, i.e., formation of parasite peptide – MHC class I complexes does not involve proteasomal cleavage (41).
- Proteins secreted via the classical route or located on the surface of the parasite are more efficiently presented to stimulate CD4 and CD8 T cells (40, 42, 43).
- The major antigen-presenting cells initiating the immune response are dendritic cells (44, 45) while infected macrophages are likely the most frequent antigen-presenting cell during infection (46, 47).
- Macrophages need to be activated, e.g., through cytokines such as IFN-γ to express MHC class II molecules, a prerequisite to present antigens to CD4 T cells (48, 49).
- Only a minority of infected macrophages seems to interact with *Leishmania*-specific T cells *in vivo* (46).

## Bullet Point Style Summary of Processes and Molecule Numbers Relevant for Antigen-Presentation

- Mature dendritic cells express up to 10^6^–10^7^ MHC Class II and 10^5^ MHC I molecules per cell (50, 51).
- Mature dendritic cell “fix” a surface MHC class II-peptide complex repertoire to present an immunological snap shot to interacting T cells (52).
- Activated macrophages express 10^5^–10^6^ MHC Class II and 10^5^ MHC I molecules per cell and these are undergoing turn over and recycling (53).
- Immature dendritic cells and Macrophages constantly cycle MHC–peptide complexes from cell surface through endocytic peptide loading enabling compartments back to the surface allowing peptide sampling over time (54).
- Cells display two populations of MHC–peptide complexes, one with a fast off rate of the peptide ligand and one with slow off-rates, a property that in combination with dynamic sampling is a mechanism to enrich for the thermodynamically most stable MHC–peptide complexes for presentation (55).
- Estimates of the number of cognate MHC–peptide complexes required for successful T cell stimulations vary from a single complex (56) to several hundred (57) and a number in the order of 10^2^ is a reasonable estimate (58).
- Amastigotes yield ~2–4 × 10^−12^ g of protein per cell that corresponds to 3–5 × 10^7^ protein molecules per parasite assuming an average size of ~50 kDa per molecule (40, 59).
- *Leishmania* genomes encode some 8200 distinct proteins (60), which are predicted to encode nearly 3 × 10^5^ MHC class I epitopes with binding capacity for MHC even when only a single MHC class I allele is considered (30).
- The average number of predicted epitopes per protein is thus >36 hence >10^9^ epitope molecules are likely to be generated from a single parasite if all proteins were processed.
- Parasite proteins may become accessible for the presentation machinery either through parasite lysis, directed release (through exosomes or via classical secretion) or surface exposure and hydrolytic release.

## Algorithms of Reverse Vaccinology to Identify Candidate Proteins for Anti-*Leishmania* Vaccine Development

The most puristic Reverse Vaccinology algorithms to identify candidate vaccine antigens adapted for leishmaniasis proceeded stepwise from genome to T cell epitope prediction (28, 30). For example, Herrera-Najera et al. (30) based their algorithm on the condition that a vaccine protects through induction of CD8 T cells recognizing a parasite protein-derived epitope in the context of MHC class I molecules. In a first step, they analyzed the complete genome for encoding peptides predicted to have MHC–ligand properties (for selected mouse H-2 class I alleles) using a sliding window of 8–11mer amino acids over the entire open reading frames and adapting a filter to account for proteasome-processing preferences implemented in the RankPep software. This identified ~3 × 10^5^ candidate epitopes. To reduce this number, a stringent but arbitrary threshold of the binding score to MHC was introduced resulting in 250 candidate peptides. In step 2 of the process, these candidates were further analyzed using different T cell epitope prediction algorithms. A set of 78 epitopes was predicted by all or nearly all software. In step 3, the 78 epitopes were compared to the predicted proteomes of putative hosts based on mouse and human genome data, the rationale being to reduce the risk of inducing autoimmune reactions. In this step, it was considered satisfactory that none of the selected peptide-epitopes had >80% identity with a host protein. However, there were peptides with lower identity, i.e., with up to 9 of 11 amino acids identical. Step 4 checked for conservation of the candidate protein containing the epitope(s) in different *Leishmania* spp. and other kinetoplastids. The authors noted that their algorithm did not identify any of the known, experimentally validated candidates. These failed the arbitrarily set stringent threshold for the MHC-binding score in step 1.

An alternative algorithm based on the same idea of vaccine mode of action was developed by John et al. (28). In this case, additional characteristics of a vaccine antigen were assumed and used for filtering. In step 1, subcellular localization was analyzed using PSORT and TMHMM software, respectively, and used to enrich for 903 proteins with predictions for plasma membrane localization or secretion and with counter-selection of proteins with more than one predicted transmembrane domain. This list was purged in step 2 of proteins showing homology to murine or human host proteins leaving 553 candidates in the basket. Selection step 3 analyzed the presence of MHC class I binding and step 4 of MHC class II binding peptides using several programs. Unfortunately, the adopted thresholds that reduced the number to 19 candidates were not described. This final set was tested again for similarity to host self-epitopes but this did not reduce the number further. As before, no experimentally identified protein antigen has passed this selection process.

While both of these approaches identified potentially immunogenic epitopes [in fact immunogenicity was demonstrated in the case of Ref. (30)], the fact that these algorithms did not identify any of the experimentally tested vaccine proteins/epitopes (which is not the same as the ideal vaccine antigen) is worrisome. What is missing?

## Reflections on Improving Reverse Vaccinology Approaches for the Prediction of Candidate Antigens for a Vaccine Against Leishmaniasis

The working hypothesis that the success of a vaccine to prevent or treat VL in humans will rely on the induction of CD4 and CD8 T cells is valid. However, individual steps in the algorithms aiming at antigen identification need to be scrutinized on the one hand for the validity of underlying concepts and logic and on the other hand for their effectiveness as selecting filters. Since the abovementioned studies offer recent examples, I shall follow steps as proposed in their algorithms for illustration.

Herrera-Najera et al. (30) started with predicting MHC-binding peptides considering the proteasomal pathway of peptide generation. While there is evidence against involvement of the proteasome for cross-presentation of parasite-delivered antigens (41), there is currently no evidence in support of it. Thus, this filter may neither be necessary nor instructive. MHC-binding peptide prediction highlighted nearly 3 × 10^5^ candidates. Thus, every ORF is likely to encode more than one candidate hence the filter lacks efficiency. An arbitrary threshold as introduced can seemingly provide filtering capacity but will quickly become too stringent since in the said example it excluded all experimentally identified candidates. The next step involved selection based on T cell epitope predicting algorithms. This filtering is highly error prone and probably superfluous as the T cell receptor is an explorative, adaptive molecule that can recognize epitope variants (61). Because of this, the advantage of implementing this step can be questioned. In addition, there is little evidence from many other areas of its predicting power.

Both *in silico* Reverse Vaccinology algorithms discussed added then an additional step of counter-selection at the epitope stage by testing for molecular mimicry of proteins of putative host species. In theory, this is totally reasonable. In practice, this is either insensitive [see Ref. (30)] or seems impossible since cognate interaction of MHC–peptide complexes with TCRs is not as specific as previously thought and, where analyzed, the sequence space allowing mimicry is extensive (62). The intricacies of this have been reviewed recently in the context of cancer-cell specific epitopes and provide instructive insight (63). In conclusion, T cell epitope prediction may have no and selection against host proteins very limited practical value.

Does this mean that genome and other genomics information offers no opportunities of adapting the Reverse Vaccinology approach to our field? This view may be too pessimistic. The algorithm proposed by John et al. (28) enriched for proteins predicted to be secreted or surface localized. Reasons for this are that these two topologies will facilitate access for the MHC processing and loading machineries from living, actively replicating parasites. This assumption is founded on experimental evidence since phagocytes infected with parasites genetically engineered to secrete or surface expose trackable antigens were more readily presenting the antigens (40, 42). However, evidence that this situation is the prevailing or most relevant mode of antigen-delivery for presentation *in vivo* is still scarce. In fact recent data from *in vivo* tracing approaches suggest that control of infection and healing involves engagement of only a minority of infected or parasite-exposed cells with protective T cells (47, 64). Also, there is evidence that major normally secreted antigens are relatively resistant to proteolytic processing, as shown for the highly abundant secreted proteophosphoglycans of *Leishmania mexicana* (65). This is probably no surprise since parasite products secreted into the phagolysosomal compartment should have evolved this property to preserve their function. Nonetheless, under the assumption that a productively infected cell is the most relevant antigen-presenting cell in these infections, filtering candidate antigens for secreted or surface exposed localization remains reasonable.

Alternative scenarios of antigen-presentation that should be considered are host cells that become activated under pro-inflammatory conditions to kill the parasites or cells in which a fraction of parasites may undergo spontaneous lysis, e.g., due to faulty replication. In these cases, the entire set of parasite proteins will ultimately become available for processing and presentation. Of note, from the point of view, which set of proteins will be presented these modes are also akin to a scenario where antigens reach the processing machinery via the recently proposed secretion pathway involving exosome release by live parasite. Antigens accessible to the processing machinery in these situations are similar because the proteome of exosomes largely overlaps that of the abundant protein set present in whole parasite lysates [e.g., compare data from Ref. (39, 66)].

In all these situations, I would argue that relative protein abundance is the single most important parameter for candidate antigen selection and is of a high practical value. The algorithms discussed so far did not take relative protein abundance into account. Instead they assumed equivalence of all predicted proteins with respect to their chance to being successfully processed and loaded onto MHC molecules. Not having considered abundance may be an additional reason why none of the experimentally identified candidate antigens were within the set identified by purely bioinformatics approaches. Fortunately proteome data sets reporting about relative abundance of proteins are available and these resources are permanently expanding (67–73) although improvements to the reporting of quantitative aspects of proteome data would be desirable.

**Figure 1:**
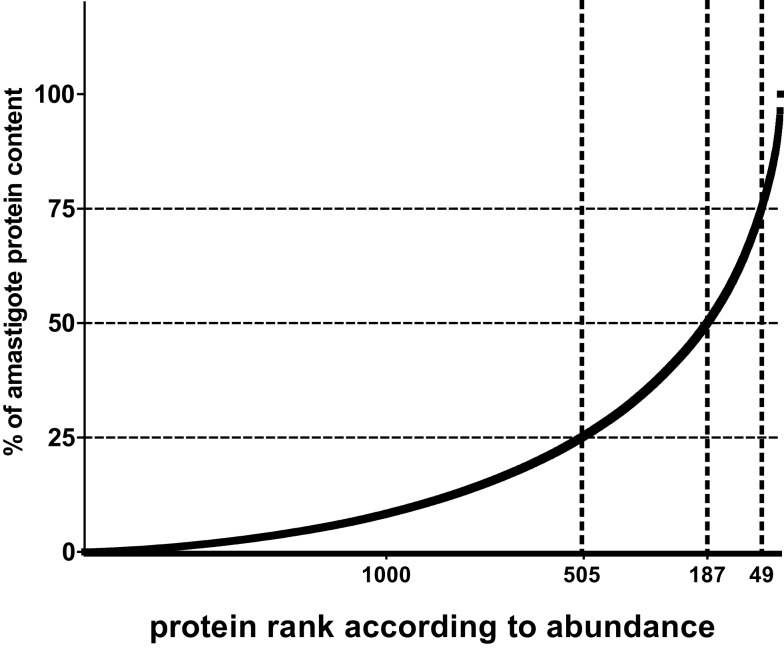
**Relative contribution of individual proteins to total protein content of *Leishmania* amastigotes**. For illustration, the contribution of each of 1764 proteins detected by shot gun proteomics in *L. mexicana* amastigotes (66) is expressed as percent to total protein mass and values plotted in ordered fashion for each protein. Numbers on *X*-axes show the rank of the *n*th protein at the thresholds of 75, 50, and 25% of total mass.

In the following, I would like to analyze the potential of integrating quantitative proteome information with a quantitative view of the presentation process (see also bullet point style summaries above) into an algorithm of Reverse Vaccinology. If we accept that in principle each parasite protein contains functional MHC I and II binding peptides and, thus, potential T cell epitopes, we may simply base our estimates on the number of protein molecules per parasite (~5 × 10^7^ molecules). Similarly, if we agree that both CD4 and CD8 cells are relevant for protection, we can base our analysis on the number of MHC class I molecules expressed per antigen-presenting cell (~10^5^ molecules) since this is thought to be lower than the number of class II molecules, hence can be considered the limiting peptide receptor species. To illustrate the next steps, I will base my arguments on a data set published by our group. We aimed at identifying the relative abundance of proteins in amastigotes of *L. mexicana* based on a label free method that deduces a protein abundance index (emPAI) for each protein in a data set (66, 74). The reason for this choice is simply that equivalent data is not easily accessible in other comparable proteome data sets. When parasite proteins are ranked according to their emPAI value, it is quickly realized that proteins encoded by less than 50 and 200 genes contribute more than 25 and 50% of the total parasite protein content in terms of mass (Figure 1).

**Figure 2:**
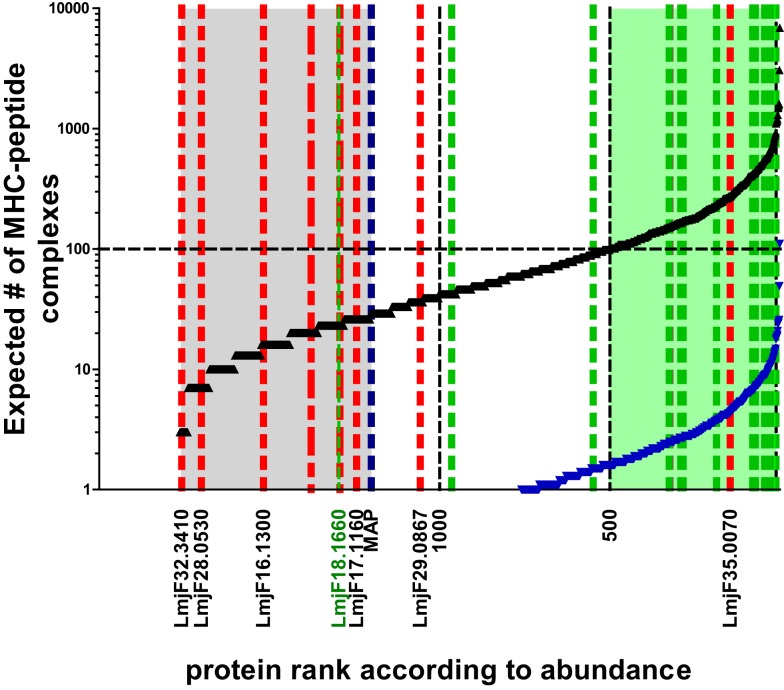
**Expected number of individual MHC–peptide complexes depending on protein abundance**. The black curve indicates expected number of complexes assuming protein copy number is most determining. Blue curve indicates lower boundary of the model basing expected number of complexes on the assumption that all protein are first degraded to peptides. The likelihood of complex formation for a peptide derived from the average size protein (52 kDa) is thus reduced 36-fold [i.e., the average number of predicted epitopes deducted from Herrera-Najera et al. (30)]. Green shaded area in plot: proteins above the threshold of 100 MHC–peptide complexes when sampled by 10^5^ MHC molecules assuming one binding peptide per protein. Area shaded in gray: proteins with ranks below that of lysosomal membrane acid phosphatase (MAP; blue dotted line) for which the corresponding molecule number per parasite was experimentally shown to be non-stimulatory for T cells. Green dotted lines indicate ranks of proteins with experimental evidence of T cell recognition (in ascending order GRP78, HSP83, Histone H-2, STI-1, CSP-B, Glu synthetase, ATP synthase, LACK, LeIF, TSA, gp63, KMP-11, HSP20, 60S ribosomal protein, nucleoside hydrolase, amastin, SMT, and γ-glutamylcysteine synthetase = LmjF18.1660). Blue dotted line indicates lysosomal membrane acid phosphatase (MAP) for which the corresponding molecule number per parasite was experimentally shown to be non-stimulatory for T cells. Red dotted lines refer to the rank of proteins identified *in silico* to contain candidate epitopes by Herrera-Najera et al. (30) (again in ascending order, LmjF35.0070, LmjF29.0867, LmjF17.1160, LmjF16.1300, LmjF28.0530, and LmjF32.3410), or John et al. (28) (red stippled line: PI-3 Kinase like protein, lipase).

These relative values can be expressed as number of molecules per parasite taking into consideration the respective molecular weight and the total protein content per parasite (~4 pg). Thus, the copy number per cell of proteins detected in current proteomic analyses ranges from a few million to a couple of 100 molecules. MHC–peptide complex formation, however, ultimately follows the law of mass, hence abundant molecules have a greater chance of becoming processed and ensuing peptides bound to the MHC-binding groove. The simplest version of predicting the chance of a protein to be successful in this respect is to calculate an expected value for how often this may be the case if 10^5^ MHC molecules are allowed to dip into the compartment where the peptides are formed and pick a peptide (remember as a further simplification, we equal 1 protein to 1 epitope). The expected value of MHC–peptide complexes for each protein in the data set can be plotted in an ordered way according to protein abundance, which produces an S-shaped curve (Figure 2). For candidate prediction purposes, it is then necessary to try to define rationally a threshold below which the chance of a peptide species to be bound by a stimulatory number of MHC molecules becomes negligible. One way to set this threshold is to adopt the number of surface MHC–epitope complexes required for stimulating T cells as defined by immunologists. As mentioned before a reasonable estimate for this is in the order of 100 complexes, which is indicated by a horizontal line in Figure 2. The expected number of MHC-peptides was calculated for experimentally validated, naturally immunogenic proteins and, indeed, for the majority the expected number is above this threshold (Figure 2; green shaded area of plot). A complementary way to define the threshold is by extrapolation of experimental data on individual parasite proteins that were assayed either in vaccination studies or in T cell stimulation tests. Importantly, there is experimental evidence for a lower boundary of the protein copy number per cell value at which infected macrophages do no longer stimulate the respective antigen-specific CD4 T cells (40). This threshold is indicated as a blue dotted line in Figure 2.

The presented approach is easily expanded or adapted to additional proteomic data sets when information on relative protein abundance becomes available. It reveals not only the likely reason why most experimentally studied antigens were immuno- and antigenic but also defines a large number of additional candidates. In contrast, the majority of the candidates predicted purely by bioinformatics (28, 30) were not in the proteome data set. This may indeed indicate that their respective copy number per parasite was below detection levels of the method (which is then likely to be also below the detection level of the MHC presentation machinery). However, this conclusion has to be drawn with caution as the likelihood of detecting the protein by proteomics can be reduced for technical reasons, which is the case, e.g., for integral membrane proteins [see also Ref. (66)]. The latter however can be reasonably well-predicted through bioinformatics analysis.

Of course an algorithm as presented above, that integrates protein abundance to derive the set of likely immunogenic and hence vaccine candidate proteins, is simplistic. But, its advantages are its practical value and high flexibility since any change in parameters can be easily accommodated. Changing parameters will essentially only re-position the threshold value for the effective number of MHC–peptide complexes. For example, the threshold may change if dynamic sampling of the peptide pool by recycling MHC is integrated over the time of an infection cycle. In this case, peptide off-rates from MHC–peptide complexes may be a valuable, bioinformatically accessible factor to improve the algorithm. It has been shown that kinetic stability of MHC–peptide complexes is probably the single most important determinant that defines immunodominant T cell epitopes (75). Furthermore, dynamic exchange of weakly binding peptides for more stably bound peptides has been shown to occur upon MHC-peptide recycling from and to the plasma membrane (76). Thus, in theory the algorithm for ranking candidates may include a weighting factor based on predicted peptide off-rates from their MHC receptors. This factor may be multiplied by protein/peptide abundance to derive an “effective concentration” of a particular peptide. A high effective concentration may be the reason underlying the efficacy of leishmanial γ-glutamylcysteine synthetase as an effective vaccine in animal models of *Leishmania donovani* infection (77, 78). Alternatively, this antigen may be more abundantly expressed in *L. donovani* than suggested by the data derived from *L. mexicana* that were used here for illustration. Consistent with the latter idea, the same γ-glutamylcysteine synthetase-based vaccines were less effective against *L. mexicana* (79). Unfortunately, experimental data on an exemplary set of antigens to derive such a weighting factor are lacking and given the uncertainties associated with MHC-peptide ligand predicting algorithms the practical value of such a factor is currently difficult to assess.

In summary, developing an algorithm to adapt Reverse Vaccinology for the identification of antigens for anti-VL vaccine should include as a first step quantitative aspects of protein expression and incorporate the growing resource of proteomic data sets. On its own, however, this approach still leaves one with some 500 candidates. Selection against epitopes with homology to host proteins is certainly advisable but one should be aware of its limitations and the gargantuan dimension of its unknowns due to the fact that T cells recognize a sequence space (63). If adopted, the definition of the immunological self should probably include commensals (80). Thus, selection against peptides with homology to host proteins seems on the one hand not rigorous enough and, on the other hand, appears to adopt a functionally limited if not wrong concept of self. Nonetheless, integration of this information and data on predicted candidate antigen localization, MHC-peptide stability, conservation between parasites and selection of genus-specific antigens may all be criteria of practical value. It should be noted though that the latter two are common sense criteria but there is scarcely any experimental data (81) to validate them.

## Conflict of Interest Statement

The author declares that the research was conducted in the absence of any commercial or financial relationships that could be construed as a potential conflict of interest.
